# List of Licentiates

**Published:** 1877-12

**Authors:** 


OFFICIAL LIST of Licentiates of the Illinois State Board of Health who have qualified since the last announcement made in this JOURNAL:

### COOK COUNTY.

| | | |
|---|---|---|
| Burrell, C. W | | Chicago Medical College, March 12, 1872 |
| Clark, A. B., Chicago | | Chicago Medical College, March 21, 1876 |
| Byford, Henry T., Chicago | | Chicago Medlcal College, March 13, 1873 |
| Park, Roswell, | " | Chicago Medical Chicago, March 21, 1876 |
| Dyas, Geo. K. | " | Chicago Medical College, March 21, 1869 |
| Bottomley, B. H. | " | Medical Department, Lind University, March 3, 1863 |
| Sterl, Alexander, | " | University of the City of N. Y., March 5th, 1866 |
| Braun, Louis, | " | Rush Medical College, Feb. 15, 1876 |
| Smith, A. W., | " | Eclectic Medical Institute, Cin. O., May 21, 1872 |
| McElray, P. H., | " | Rush Medical College, Feb. 1, 1871 |
| Seiffert Rudolph, | " | University of Vienna, Austria, Feb. 13, 1852 |
| Hutchinson, Jas. M. | " | Chicago Medical College, March 3, 1867 |
| Hooper Henry | " | Harvard Medical College, June, 1869 |
| Cole, Harlan P., | " | N. Y. Homeœopathic Medical College, March 3, 1872<br>Chicago Medical College March 14, 1871 |
| Johnson, H. A., | " | Rush Medical College, Feb., 1852 |
| Williams, T. D., | " | Hahnemann Medical College, Chicago, Feb. 23, 1871 |
| Fellows, H. B., | " | Western Homeo College, Cleveland, March 1, 1861 |
| Ogden, E. J., | " | The University of the City of N. Y. March 9, 1855<br>The Victoria College, Coburg, Canada, July 5, 1855 |
| Ogden, Milton D., | " | The Hahnemann Medical College, Chicago, Feb. 28, 1862 |
| Wilder, F. M., | " | The University of Michigan, March 25, 1868 |
| Dexter, Ransom, | " | The University of Michigan, March 26, 1862 |
| Copp, B. D., | " | Rush Medical College, Feb. 1, 1871 |
| Henrotin, F., | " | Rush Medical College, Feb. 1, 1868 |

Degeller, John, " ..............The University of Munich, Bavaria, June 9, 1864
Dovelittle, W. H. " ..........................Rush Medical College, Feb. 15, 1876
Root, D. S., " ........: .......... .....Rush Medical College, Jan. 25, 1867
Hall, Robt. T., " ..........................Rush Medical College, Jan. 17, 1872
Ware, Lyman, " .....................Chicago Medical College, March 1, 1866
Wanzer, Hiram, " .................The Lind University, Chicago, March 12, 1861
Henry, C. P., " ........... .....The University of Berlin, Prussia, July, 1859
Flood, John, " .........Bennett Elec Medical College, Chicago, Jan. 26, 1873
Jenner, Herman, " ..............The University of Munich, Bavaria, March 6, 1875
Twining, S. D., " ..........The Medical Department, Yale College, Jan. 14, 1864
Hunt, William C. " ....... ....................Rush Medical College, Feb. 22, 1852
Bluthardt, T. J. " .................The Lind University, Chicago, March 4, 1861
Baldwin, H. N., " ..........Hahnemann Medical College, Chicago, Feb. 22, 1877
Kelly, F. W. " ....................The Medical Dep't University of Vermont, ................Burlington, Vt., March, 1860
Baker, Sibelia F. Chicago....Female Medical College, Philadelphia, Pa., March 12, 1870
Duncanson, A. A.. " ....Western Homeopathic Med. Coll. Cleveland, Mar. 10, 1858
Drake, Thos. B., " ...........Albany Medical Collega, Albany, N, Y., June 7, 1857
Young, Henry N., " .........Jefferson Medical College, Philadelphia, March 8, 1856
Stockham, G. H., " ....Eclectic Medical Institute, Cincinnati, Ohio, March 2, 1856
Taylor, W. W., " ...... ............University of Louisville, Ky., March 31, 1871
Fisher, John C., " ..Long Island College Hospital, Brooklyn, N. Y., June 22, 1876
Fredigke, J. G., " ......................Chicago Medical College. March, 5, 1867
Dodge, Lewis, " ..Homeopathic Medical College of Pennsylvania, March 2, 1850
Bailey, Samuel G., " ........Jefferson Medical College, Philadelphia, March 20, 1844
Fischer, Gustavus " ..................University of Prague, Bohemia, April 1, 1871
Willard, Geo. E., " .......................Chicago Medical College, March, 10, 1874
Wolgamott, Geo. W.," ...............University of Iowa, Keokuk, Iowa, Feb. 21, 1861
Strong, Albert B., " ........................Rush Medical College, Jan. 17th, 1872
Taylor, Theo. T., " ........Kentucky School of Medicine, Louisville, March 1, 1869
Martin, William, " ............. ..........Chicago Medical College, March 5, 1867
Roler, E. O. F., " ............................ Rush Medical College, Feb. 16, 1859
Hulett, S. Eugene, " ...........Hahnemann Medical College, Chicago, Feb. 10, 1876
Kerler, C. L., " ..........University of Vienna, Vienna, Austria, April 23, 1854
Adama, Andrews, " ..........The University of Heidleberg, Germany, May 4, 1856
Chapman, F. M., " ................Bennett Eclectic Medical College, Feb. 21, 1877
Bassett, George R., " .................Bennett Eclectic Medical College, Feb. 21, 1877
Champlin, A. H., " .... .................The University of Michigan, March 31, 1869
Heymann, H., " .........................University of Bonn, Prussia, Aug. 18, 1860
Nelson, Daniel T., " ........ ...................Harvard Medical College, March 8, 1865
Chaffec, John B., " ...........McDowell Medical College, St. Louis, March 20, 1861
Otto, Joseph, " ......... ..........................Rush Medical College, Jan. 25, 1865
Hempstead, Chas. W., Chicago ...............University of Pennsylvania, April 2, 1852
Ulrich, Julius, " .............University of Vienna, Austria, Aug. 15, 1847
Peak, Orin, Oak Park.................... ..........University of Michigan, March 30, 1854
Tucker, Dexter, M., Chicago............................Harvard Medical College, March 4, 1852
Otto, Joseph P., Irondale..........................Chicago Medical College, March 13, 1873
Jewell, J. S., Chicago...........................Lind University, Chicago, March 11, 1860
Hathaway, R.W., " ....................Syracuse Eclectic Medical College, March, 1850
Hobart, H. M., " ..............Hahnemann Medical College, Chicago, Feb. 10, 1876
Blanchard, Wallace, Chicago.......................Chicago Medical College, Ma ch, 1869
Gibbs, Lucius O., " .............Bennett Eclectic Medical College, May 20, 1872
Dyas, W. G., Chicago...............The Royal College of Surgeons, Dublin, Jan. 7, 1845
Quinlan, C. H., " ........... ......................Rush Medical College, February, 1865
Van Doozer, B. R., Chicago.......................Chicago Medical College, March 12. 1868
Piero, F. L., " .......University of Vermont, Burlington, Vt., May 15, 1866
Mauro, Andrea, " .............University of Naples, Naples, Italy, Sept. 5, 1861
Bate, John, " .............Bennett Eclectic Medical College, March 2, 1876

Davies, W. H., " .....The Eclectic Medical College, Cincinnati, Feb. 14, 1865
Dodge, David, " ..............The Columbian College, D. C., March 2, 1841; ........................Rush Medical College, Jan. 20, 1871
McCarthy, William " ......... The McGill Medical College, Montreal, May 3, 1687
Meloney, William B., " .....The University of Michigan, Ann Arbor, March 20, 1864
Morey, A. C., " .....Jefferson Medical College, Philadelphia, March, 7, 1857
Skelton, Elizabeth D., " The Woman's Hospital Med. College, Chicago, March 2, 1875
.Hurlbut, H. N.. " Licentiate of Ashtabula College, Ohio Med, Soc., Feb. 25, 1331; The Willoughby University, Willoughby, O., Feb. ..........................................20, 1840
Bottsford, Harriette A., " ......Female Medical College, Philadelphia, March 12, 1875
Davies, Charles G., " ....The University of Virginia, Charlottsville, July 3, 1873; ..............and Missouri Medical College, March 11, 1874
Fry, Isaac H., " ..........Chicago Medical College, Chicago, March 21, 1876
McArthur, R. D., " ..........McGill University, Montreal, Canada, May 3, 1867
Ely, Charles Franklin, " N. Y. Homeopathic Medical Coll., N. Y. city, March 11, 1877
Harcourt, Luke A., " ....................University of Buffalo, N. Y., Feb. 25, 1868
Wisner, Sarah D., " Hahnemann Medical College, Chicago, Chicago, Feb. 4, 1877

OTHER COUNTIES.

Chapman, P. E., Sigel, Shelby..................Louisville Medical College, Feb. 28, 1873
Dunn, L. D., Moline, Rock Island......................Rush Medical College, Feb. 18, 1857
Klingberg, A., Moline, Rock Island.....................Rush Medical College, Feb. 3, 1869
Morey, J. W., Moline, Rock Island..............Rush Medical College, January 25, 1867
Davison, J. B., Moline, Rock Island.....Jefferson Medical College, Phila., March 7, 1868
Ellis, D. E., Belvidere, Boone......Geneva Medical College, Geneva, N. Y., Jan. 24, 1843
Scott, Charles, Belvidere, Boone................Rush Medical College, February 16, 1865
Ehrlich, H. R., Lincoln, Logan.....College of Phy. & Sur., Keokuk, Iowa, Feb. 20, 1872
Miller, George H., Galena, JoDaviess............Rush Medical College, February 17, 1874
McPherson, J. B., Gilman, Iroquis....................University of Edinburgh, Scotland
Brockhart, Louis, Majority Point, Cumberland................Rush Medical College, 1858
Eskridge, J. H., Majority Point, Cumberland...Rush Medical College, February 15, 1876
Trabue, J. W., Shipman, Macoupin..............Rush Medical College, February 21, 1855
David, Cyrenius A., Sheridan, LaSalle............Rush Medical College, February 3, 1869
McCaudless, W. L., Perry..........................Rush Medical College, January 17, 1872
Merrick, G. C., Manteno, Kankakee.............Rush Medical College, February 20, 1851
Mitchell, R. J.. Girard, Macoupin..................Rush Medical College, February 1, 1871
Macklin, George M., Waterman, DeKalb..........Rush Medical College, February I, 1871
Jay, Hollis, Makena, Will.............Cincinnati Eclectic Medical College, April 4, 1859
Johnson, Wm. C., Springfield, Sangamon.........Rush Medical College, February 3, 1869
Green, George, Bristol, Kendall..................Rush Medical College, February 2, 1870
Ralph, John B., Rock Falls, Whiteside..........Rush Medical College, February 3, 1869
Ray, Robert B., Fairmount, Vermilion,..........Rush Medical College, February 15, 1860
McVey, Rich'd E., Waverly, Morgan............Rush Medical College, February 25, 1861
Morse, Simon P., Cambridge, Henry............ Rush Medical College, February 2. 1870
Kinnear, A. H., Metamora, Woodford............Rush Medical College, January 27, 1864
Koch, Charles L., Quincy, Adams...............Rush Medical College, February 27, 1877
Kerr, Charles, Pawnee, Sangamon...............Rush Medical College, January 25, 1865
Bartlett, A. I., Virden, Macoupin..................Rush Medical College, February 4, 1862
Barry, E. L. H., Jerseyville, Jersey...............Rush Medical College, February 15, 1860
Brown, Wm. C., Geneseo, Henry..................Rush Medical College, February 25, 1861
Wilson, Benj., Chambersburg, Pike..............Rush Medical College, February 18, 1857
Clarke, Wesley, Dix, Marion........................Rush Medical College, January 25, 1867
Wheeler, M. S., Auburn, Sangamon............ .Rush Medical College, February 5, 1868
Elder, S. S., Farm Ridge, LaSalle.................Rush Medicai College, January 25, 1865
Cochran, W. G., Farmer City, De Witt...........Rush Medical College, February 3, 1869
Clark, A. L., Elgin, Kane....Eclectic Medical Institute, Cincinnati, O., February 6, 1861
Harris, H. L., Fossland, Champaign.............Rush Medical College, February 16, 1875
Hurd, D. L., Earlville, LaSalle...Bennett Ecclectic Med. College, Chicago, May 26, 1870

Newkirk, G., Washburn, Woodford...............Rush Medical College, February 5, 1868
Taylor, John J., Streator, LaSalle.................Rush Medical College. January 25, 1867
Tebo, George H,, Mt. Sterling, Brown............Rush Medical College, January 17, 1872
Tweddale, James, Wasuburn, Woodford ........Rush Medical College, February 3, 1869
Smith, E. R., Edgington, Rock Island...... ...Rush Medical College, February 15, 1876
Stetson, J. R., Sheffield, Bureau.................. Rush Medical College, February 2, 1870
Drennan, D. A., Springfield, Sangamon..........Rush Medical College, February 16, 1875
Farrar, L. B., Paxton, Ford..................Berkshire Medical College, November 8, 1848
Farrar, Laura E., Paxton, Ford..............Hahnemann Medical College, Chicago, 1872
Pomeranc, Marx, Huntley, McHenry..The University of Wirceberg, Bavaria, Apl. 1, 1866
Johnson, J. P., Peoria, Peoria.....................The Ohio Medical College, March 1, 1859
Lucas, B. C. K , Peoria, Peoria.....The Long Island Col. Hosp., Brooklyn, June 24, 1875
Brown, J. L., Peoria, Peoria.........Ohio Medical College, Cincinnati, O., March 2, 1868
Mitchell, J. A., Peoria, Peoria....Jefferson Medical College, Philadelphia, March 10, 1872
Lucas, George L., Peoria, Peoria..........Jefferson Medical College, Phila., March 6, 1852
Du Mars, R. A., Peoria, Peoria.........Med. Dept. University of Penna., March 12, 1876
Ballou, N. E. Sandwich, DeKalb.............. ...Berkshire Medical College, Nov. 10, 1846
Gatchell, H. P., Jr., Highwood, Lake.. ...Hahnemann Med. Col., Chicago, Feb. 3, 1875
Lingood, John R., Rossville, Vermilion...............University of Penna., March 13, 1873
Knapp, James B., Bardolph, McDonough..................University of Buffalo—no date
Molitor, Nickolaus J., Somonauk, DeKalb.............Rush Medical College, July 1, 1869
Barnett, Charles F., Bunker Hill, Macoupin......Rush Medical College, January 21, 1863
McPherson, M. C., Polo, Ogle.......................Rush Medical College, January 17, 1872
Secord, James K., Elmwood, Peoria.......... ....Rush Medical College, January 25, 1867
Dafoe, P. V. R., Elmwood, Peoria........Victoria Med. Col., Ont., Canada, March 4, 1864
Thompson, Wm., Elmwood, Peoria..Bellevue Hosp. Med. Col., N. Y. City, March 1, 1874
McLean, John, DuQuoin, Perry......................Rush Medical College, January 1, 1863
Lake, Leonard L., Belvidere, Boone..............Rush Medical College, February 18, 1847
Williamson, George, Belvidere, Boone............Rush Medical College, February 3, 1869
Felker, John B., Amboy, Lee......................Rush Medical College, February 15, 1860
Sikes, H. B., Bardolph, McDonough......... ......Detroit Medical College, July10, 1872
Saunders, R. A., Galesburg, Knox..............St. Louis Medical College, March, 1849
Gibson, Robert, Alton, Madison..................Missouri Medical College, March 4, 1875
Rafferty, T. A., Robinson, Crawford..................Ohio Medical College, March 1, 1869
Thomson, Jno. S., Robinson, " ..................Rush Medical College, Feb. 19, 1873
Ferguson, S. T., Minooka, Grundy .................... " " " Jan. 25, 1865
Henry, Robt. F., Princeville, Peoria.................. " " " Feb. 16, 1853
True, Chas., Chatsworth, Livingston... ............. " " " Jan. 24, 1865
Holgate, James, Castleton, Stark ..................... " " " Feb. 3, 1869
Wright, Nehemiah, Chatham, Sangamon............ " " " Jan. 25, 1865
Woods, D. L., Streator, LaSalle.......................... " " " Feb. 5, 1868
Chenoweth, C., Decatur, Macon.......................Rush Medical College, Feb. 3, 1869
Chenowith, W. J. " " ..................University of Louisville, March 3, 1853
Stephens, B. J., Mt. Morris, Ogle,......................Rush Medical College, Feb. 7, 1850
Marshall, N. R. Clifton, Iroquois......................Rush Medical College, Jan. 26, 1867
Ross, B. F., Cobden, Union.......................... ..Rush Medical College, Feb. 17, 1858
Donaldson, H. C., Morrison, Whiteside..............Rush Medical College, Feb. 20, 1831
Brumagim, R. J., Morrison, Whiteside.........Castlebon Medical College, Oct. 25, 1846
Taylor Samuel, Morrison, Whiteside...........Cleveland Medical College, Feb. 22, 1844
Richings, Chas. H. Rockford, Winnebago..........Rush Medical College, Feb. 12, 1849
Kreider, Wm. L., Aaron, McDonough................Rush Medical College, Feb. 16, 1859
Kreider, Henry W., Aaron, McDonough..............Rush Medical College, Feb. 20, 1856
Davis, A. P., Paris, Edgar.......... ............ ...Rush Medical College, Jan. 25, 1867
Ramsey, Geo. W., Putnam Co..........................Rush Medical College, Feb. 15, 1876
Dubler, W. H., Windsor, Shelby.......................Rush Medical College, Jan. 25, 1865
Brunk, Chas. H., Windsor, Shelby........... ........Rush Medical College, Jan. 25, 1865
Hilsabeck, W. F., Windsor, Shelby....................Rush Medical College, Jan. 17, 1872
Thompson, S. C., Cedarville, Stephenson............Rush Medical College, Jan. 17, 1872

